# Risk of Global External Cereals Supply under the Background of the COVID-19 Pandemic: Based on the Perspective of Trade Network

**DOI:** 10.3390/foods10061168

**Published:** 2021-05-23

**Authors:** Chao Zhang, Yanzhao Yang, Zhiming Feng, Chiwei Xiao, Tingting Lang, Wenpeng Du, Ying Liu

**Affiliations:** 1 Institute of Geographic Sciences and Natural Resources Research, CAS, Beijing 100101, China; zhangc.18b@igsnrr.ac.cn (C.Z.); fengzm@igsnrr.ac.cn (Z.F.); xiaocw@igsnrr.ac.cn (C.X.); langtt.19b@igsnrr.ac.cn (T.L.); duwp.18b@igsnrr.ac.cn (W.D.); liuy.20b@igsnrr.ac.cn (Y.L.); 2 College of Resources and Environment, University of Chinese Academy of Sciences, Beijing 100049, China; 3 Faculty of Geographical Science, Beijing Normal University, Beijing 100875, China; 4 Key Laboratory of Carrying Capacity Assessment for Resource and Environment, Ministry of Natural Resources, Beijing 100101, China

**Keywords:** food security, global cereals trade, complex network analysis, external supply risk, the COVID-19 pandemic

## Abstract

International food trade is an integral part of the food system, and the COVID-19 pandemic has exposed the fragility of external food supplies. Based on the perspective of cereals trade networks (CTN), the pandemic risk is combined with the trade intensity between countries, and an assessment model of cereals external supply risk is constructed that includes external dependence index (EDI), import concentration, and risk of COVID-19 from import countries index (RICI). The results show that: (1) the global main CTN have typical scale-free characteristics, and seven communities are detected under the influence of the core countries; (2) about 60%, 50%, and 70% of countries face risks of medium and above (high and very high) external dependence, concentration of imports, and COVID-19 in the country of origin, respectively. Under the influence of the pandemic, the risk of global external cereal supply index (RECSI) has increased by 65%, and the USA-CAN communities show the highest risk index; (3) the countries with a very high risk are mainly the Pacific island countries and the Latin American and African countries. In addition, Japan, Mexico, South Korea, and 80% of the net food-importing developing countries are at high or very high RECSI levels. Approximately 50% of countries belong to the compound risk type, and many export countries belong to the RICI risk type; (4) global external food supply is subjected to multiple potential threats such as trade interruption, “price crisis”, and “payment dilemma”. The geographical proximity of community members and the geographical proximity of the pandemic risk is superimposed, increasing the regional risk of external food supply; and (5) this study confirms that the food-exporting countries should avoid the adoption of food export restriction measures and can prevent potential external supply risks from the dimensions of maintaining global food liquidity and promoting diversification of import sources. We believe that our assessment model of cereals external supply risk comprises a useful method for investigations regarding the international CTN or global food crisis under the background of the pandemic.

## 1. Introduction

Maintaining and improving food security is one of the major global challenges in the 21st century [1]. The COVID-19 pandemic (hereinafter “the pandemic”) has exposed the fragility of the global food supply and raised the specter of the global food crisis [2]. The epidemic’s (e.g., SARS, Ebola) potential damage to the future global food system has been confirmed in previous global, regional, and local food crises [3], and the negative impact of the pandemic has spread to four dimensions of food security, including availability, access, use, and stability [4]. According to the United Nations World Food Program (UN WFP) data, the pandemic may increase the number of people affected by food insecurity to 27,180 million worldwide, which is more than twice as much as in 2019 [5]. Relevant research is mainly focused on the food supply chain [6], nutrition, and health [7,8]. Geographically, research is mainly performed for low-income food-deficit countries or countries with large food production, such as the countries in sub-Saharan Africa [9], India [10]. A report from the World Bank also provided special attention to the impact of the reduction in food production in food-exporting countries on the global food supply [11].

In the context of the growing integration of the global food system [12], international food trade is increasingly becoming an integral part of the global food system. In 2016–2018, the imported quantity of main cereals (i.e., wheat, rice, and maize) reached 389.71 million tons globally on an annual level, which is about 19% of the global production of main cereals. Trade of cereals has become an important and effective way of adjusting surpluses and deficits among countries and ensuring supply [13]. Meanwhile, international trade has increased the complexity of the global food system and may increase a country’s exposure to external disturbances. Food production, transportation, and political instability in exporting countries may affect the security of their external food supply through trade, which is especially true for small countries [14]. Interdependence between countries leads to a high degree of complexity in international trade [15]. Integration of the food system promotes the diversification of global supply risks [16] and increases the possibility for countries to be influenced by the global market [17,18]. It is, therefore, helpful to clarify the status and interdependence of different countries in the global trade network and to identify the source of their external supply risks from a perspective of the cereals trade network (CTN).

Understanding the structure of the trade network helps to understand the sensitivity of the global CTN. In addition, achieving global food security requires a better understanding of how the global food trade network connects countries through the flow of food [19]. Complex network theory is widely used in the study of the patterns of commodity flows [20,21]. Puma and his colleagues applied this method to analyze the fragility of the global food system [14]. Similarly, some scholars have studied the impacts of different types of food flows on food security around the world [15,22,23,24,25]. The study by Ercsey-Ravasz et al. pointed out that the flow of products in key hubs of the food network has a more prominent and effective impact on the entire network [26]. Several studies have shown that the influence of core countries in the network gradually increases [14]. A community structure with greater intensity of internal cooperation and competition that is formed by the core countries is pushing the world trade network toward a “robust yet fragile” configuration [14]. In this network structure, when major exporting countries limit the export during a shortage in the global food market, the trade network is more vulnerable to damage. Under the influence of globalization and regionalization of trade, trade relations among the member nodes of the same community are relatively close, and interdependence is stronger, while trade relations between the member nodes of different communities are relatively loose. The core country of the community has a significant impact on the community stability, and the spatial proximity of community members is more likely to cause a crisis in the regional trade network.

Policies restricting food trade in some exporting countries were adopted in the early stages of the pandemic, which raised concerns about the stability of the global CTN and also deepened concerns of some countries about the import concentration. If a country imports food from more countries, then the lower the import risk it faces. However, if a country imports food from a single or a few countries, the higher the import risk it faces. External supply risks have long been the focus of attention in the field of resource supply [27]. The model that combines the Shannon–Wiener Index, Herfindahl–Hirschman Index (HHI), and the Political Risk Rating of the country of origin of imports, geographic spatial distance, and other parameters is widely used in energy, food, and other fields [28,29,30].

The pandemic affected the relatively stable operation of the global trade network, highlighted the vulnerability of external supply, and exposed the risk of import concentration (i.e., HHI) and external dependence on external supply. Studies that assessed the potential impact of the pandemic on global risks of external food supply from the perspective of interdependence between countries, import concentration, and external dependence of food-importing countries are still rare.

Therefore, from the trade network perspective, this research aims to explore the risks of global external food supply and selects the main cereals as the research object. Taking into account factors such as the risk of COVID-19 in the exporting country, a model (i.e., the risk of global external cereal supply index, or the RECSI) has been established to assess the global risk of external food supply. We try to answer the following questions: (1) Which countries have a significant impact on the stability of the global cereal trade network? (2) What is the flow pattern and interdependence of the global cereal trade? (3) How have the risks of global external cereal supply changed under the influence of the pandemic? What is the difference between the dominant risk factors? What are the regional differences?

Faced with the goals and issues, this study uses the global main cereals (i.e., wheat, rice, and maize) production and trade data from the FAOSTAT (2016–2018) to perform the following work. First, we use the complex network method to analyze the characteristics of the global trade network of main cereals, identify the core nodes of the trade network and characterize the flow patterns of main cereals and inter-country dependencies within the community. Second, we construct the risk of external cereal supply model that includes risk factors such as the HHI, external dependence index (EDI), and risk of COVID-19 from import countries index (RICI). Then, we assess changes in external supply risks at global, community, and country dimensions in the context of the pandemic, followed by the analysis of the dominant factors of external supply risks. We hope to grasp the extent of the pandemic’s impact on the security of the global external food supply and provide a scientific basis for different countries around the world to respond to the pandemic’s impact on external food supply.

## 2. Methods and Data Processing

Problems such as the restriction of cereals export and the decline in transport efficiency caused by the pandemic have shocked the relatively stable operation of the global trade network and exposed the fragility of the external food supply. Risk assessment of external food supply is one of the important means of resolving a possible food crisis. In general, external supply risk consists of external dependence and import concentration. External dependence refers to the ratio of imported cereals to actual consumption. The higher the EDI of a country, the higher the supply risk.

Due to the possible trade disruption in external supply, the import concentration is another risk factor for external supply. The composition of food imports is also important for security. If food imports are well-diversified, importing countries face a lower risk of supply disruptions than if all their food imports come from a single supplier [30]. The basic HHI is used in economics to measure firm concentration levels within the industry [27]. We use HHI to assess the import concentration of food [29].

The continuous development of the pandemic has caused concern in most countries due to the turmoil in the global food market. RICI is closely related to the stability of food production and trade. Therefore, RICI becomes the external food supply of the food-importing country.

**Figure 1:**
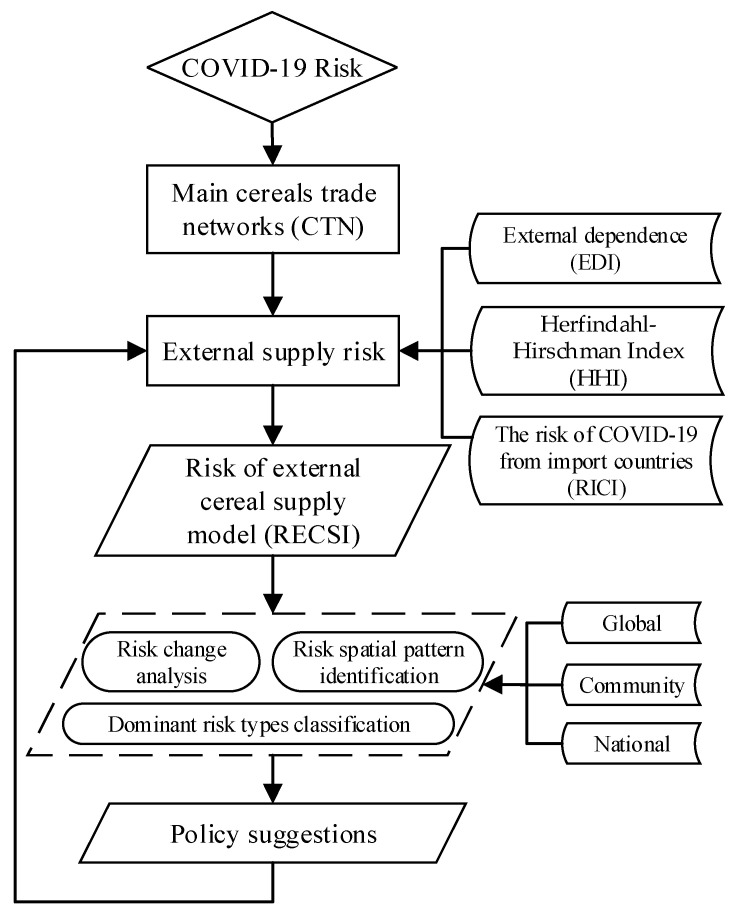
Study framework of external cereals supply risk.

Therefore, this study starts from the vulnerability of the trade network under the pandemic, builds an assessment model of the national-scale external supply risk based on EDI, HHI, and RICI, and analyzes the risk level of external food supply, identifies the risk pattern and classifies the dominant risk types from the global, community and national scales, and proposes adaptive strategies to address external supply risks. The framework of our study is presented in Figure 1.

### 2.1. Methods

#### 2.1.1. Trade Network Analysis

(1) Network properties

Node degree distribution characteristics can reflect the network shape, while different network shapes show different changes when attacked [31].

Node degree represents the number of countries that have direct trade links with the node $K_{i}$, which is defined as: $K_{i}=\sum_{j=1}^{N}a_{ij}$. In a directed network, it can be divided into out-degree and in-degree, which are defined as: $K_{i}^{in}=\sum_{j=1}^{N}a_{ji}$ and $K_{i}^{out}=\sum_{j=1}^{N}a_{ij}$. The higher the degree value, the more countries have trade links with the country and the greater the country’s influence in the trade network.

Betweenness centrality (BC) is a measure that rates the import of a node or an edge position in a network in relation to transport through the entire network, which is defined as: $BC_{i}=\frac{1}{N^{2}}\sum_{i,t}\frac{n_{si}^{i}}{g_{si}}$. In the equation, $g_{si}$ is the number of shortest paths from node *s* to node *i*, and $n_{si}^{i}$ is the number of shortest paths through node *i* in $g_{si}$ shortest paths from node *s* to node *i.* The higher the value of the betweenness centrality, the greater the importance of the nodes [32] and the greater the impact on network transmission after removing these nodes.

(2) Community Structure Detection

A community in a complex network refers to a sub-collection of nodes composed of network nodes. The community detection method can divide a network into several independent, internally connected modules to reveal the structural characteristics of the network in the community [33]. The specific calculation formula of modularity is as follows: $Q=\frac{1}{2m}\sum_{ij}(A_{ij}-\frac{k_{i}k_{j}}{2m})\partial (c_{i}c_{j})$. In the equation, $c_{i}$ and $c_{j}$ represent the communities in which the *i* and *j* nodes are located, respectively. ∂ represents binary functions for estimating whether two points are in the same community. If $c_{i}=c_{j}$, then the value is 1, otherwise the value is 0. $A_{ij}$ is the weight of the connection between nodes *i* and *j*, $k_{i}=\sum_{j}A_{ij}$ is the sum of all connection weights that contain node *i*, $m=\frac{1}{2}\sum_{ij}A_{ij}$ is the total contact weight of the entire network, and modularity is a standardized index, and the value interval is [−1, 1]. This study uses the Louvain community detection method, which is widely used to study the community structure of large networks.

This study named the community according to the country with the largest export quantity in the community. We analyze the interdependence between the flow of main cereals in the community and the interdependence between countries [34]. We divide trade relations between countries into absolute dependence (80–100%), relative dependence (60–80%), basic dependence with five levels (40–60%), important supplement (20–40%), and general supplement (0–20%). According to the proportion of import sources, trade relations between countries are divided into absolute dependence, relative dependence, basic dependence, important supplements, and general supplement in five levels.

#### 2.1.2. External Supply Risk Model Construction

Based on the analysis of the trade network structure, a model of the external cereals supply risk is constructed based on the EDI, HHI, and RICI. The details are presented below.

(1) EDI is defined as $EDI_{a}=\frac{NIQ_{a}}{DP_{a}+NIQ_{a}}$, where $NIQ_{a}$ indicates the net import quantity of country *a*, and $DP_{a}$ indicates the domestic production of the country *a*. In order to overcome the influence of negative values, the following methods are used to assign EDI values: $EDI_{a}=\{\begin{array}{ll} 1\;\;, & if\;EDI_{a}\;\leq 0\\ EDI_{a}+1 & if\;EDI_{a}\;>0 \end{array}$. Note the external dependency risk as R_EDI_, R_EDI_ ∈ [1, 2].

(2) Import concentration is characterized by HHI [35,36], which is defined as $HHI_{a}^{}=\sum_{i}{\left(\frac{IQ_{ai}}{IQ_{a}}\right)}^{2}$, where $IQ_{ai}$ and $IQ_{a}$ represent the total import quantity of country *a* and the import quantity of country *a* from country *i*, respectively. For the ease of fitting comparison, HHI_a_ + 1 represents a risk of import concentration, which is recorded as R_RICI_, R_RICI_∈ [1, 2].

(3) RICI is defined as: $RICI_{a}=\sum_{i}\frac{IQ_{\mathrm{ai}}}{IQ_{a}}\times CRI_{i}$, where $CRI_{i}$ is the COVID-19 Risk Index of country *i*. Note the COVID-19 risk from import country as R_RICI_, R_RICI_∈ [1, 2]. When there is no pandemic, the value is 1. EDI, HHI, and RICI are divided into five levels according to the natural breaks GIS method (Table 1).

Given that the above-mentioned evaluation factors are in the range of [1, 2] and have an equal impact on food security, they are all important for building a comprehensive food security risk index. This study adopts the equal interval for dividing the above-mentioned indexes into 1–10. A value of 1 to 10 is assigned in order to obtain a normalized risk factor value, and the sum method is used to calculate the RECSI: $RECSI_{a}=HHI_{a}+EDI_{a}+RICI_{a}$. According to the range of the RECSI results, the equal interval is used to divide the risk of external cereal supply into five levels (Table 1). The contribution rate of each assessment factor to the RECSI is calculated according to the proportion of R_DEI_, R_HHI_, and R_RICI_ scores in RECSI, and they are recorded as CR_DEI_, CR_HHI_, and CR_RICI_, respectively. The type of external supply risk is identified based on the contribution rate and the combination of the assessment factors (Table 2). In addition, this study records the growth rate of RECSI (or the GRECSI) before and after the pandemic as GRECSI and uses the natural breaks method to divide it into five levels (Table 1).

### 2.2. Data Processing

#### 2.2.1. Trade Network Data

Cereals trade accounts for 50% of global trade in agricultural products, more than half of direct human calorie intake, and two-thirds of feed intake [13]. This study takes as its main research object the main cereals, including wheat, maize, rice, and milled (husked rice is converted into rice, milled with a coefficient of 0.9 [37]). Trade data is from the detailed trade matrix dataset of FAOSTAT (http://www.fao.org/faostat/en/#home, accessed on 31 November 2020).

We use the import quantity to construct the main CTN. When country-specific data are missing, they can be replaced and supplemented by the conversion of import and export data. For example, when querying import data, if import data of reporter countries from the partner country is missing, we can obtain the import data of reporter countries A by querying export data of all reporter countries to country A. After data supplementation (about 5.88%), trade data of 218 countries were finally obtained. In addition, we did not set a threshold for the scale of trade flows. Population and production data come from the corresponding FAOSTAT dataset. To overcome the impact of data fluctuations, we used Excel to perform average processing on the latest FAO data (2016–2018) to characterize current global food production and trade conditions and used Gephi software to analyze network characteristics and for visualization purposes (https://gephi.org/, accessed on 5 November 2020).

#### 2.2.2. COVID-19 Risk Data

COVID-19 risk data comes from the global COVID-19 Risk Index jointly released by caixin.com (http://covid19-risk-index.com/, accessed on 1 February 2021), Fudan University, and other institutions. Data on missing countries are supplemented based on the average of their sub-regions (United Nations classification standards) in order to overcome the impact of data fluctuations. The average value from 15 August to 31 December 2020 is used.

## 3. Results

This section may be divided by subheadings. It should provide a concise and precise description of the experimental results, their interpretation, as well as the experimental conclusions that can be drawn.

### 3.1. Network Characteristics and Analysis

**Figure 2:**
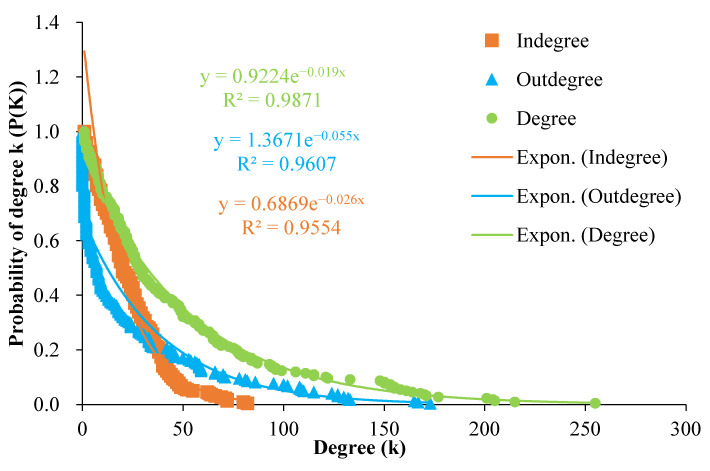
Probability distribution for the degree, in-degree, and out-degree of the export volumes of main cereals. The X-axis represents the values of the node’s in-degree, out-degree, and degree. The Y-axis refers to the probability of the corresponding value of the cereals trade. The orange, blue, and green lines indicate the exponential fitting curve of the degree, in-degree, and out-degree, respectively.

Global CTN has 218 nodes, 5118 edges, and the annual trade quantity reaches 389.71 million tons. The results of the probability distribution fitting show that the R^2^ distribution of CTN’s degree, out-degree, and in-degree are all higher than 95% and decreasing, the CTN has scale-free distribution characteristics, strong heterogeneity, and the core node has a major leading role in the CTN (Figure 2). In terms of flow, the top 20 countries in global exports accounted for 91.86% of the world’s main cereals exports, and the top 10 countries accounted for almost 80% of the exports. The top 20 countries in terms of imports account for about 60% of the world’s main cereals imports, and the top 10 countries account for about 40% of the imports (Table 3). The export network is more heterogeneous than the import network, and the importance of its core nodes is more obvious.

**Figure 3:**
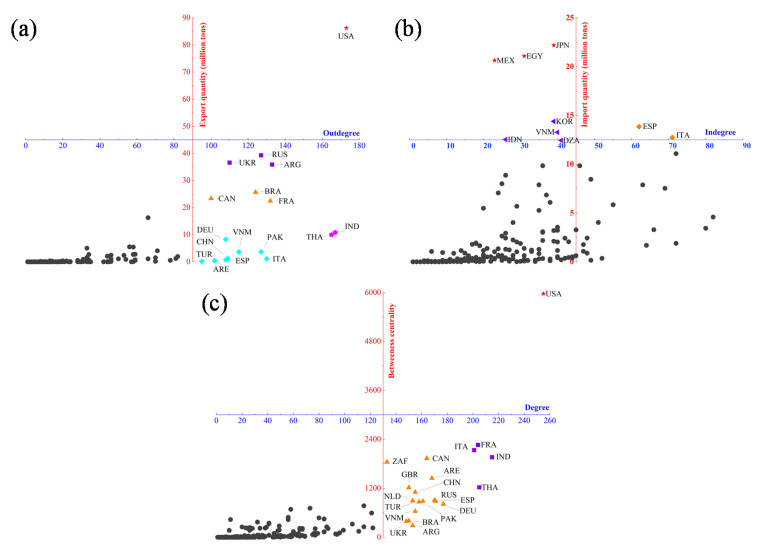
Scatter-plot of every country: (**a**) out-degree vs. export quantity; (**b**) in-degree vs. import quantity; and (**c**) degree vs. betweenness centrality. The same color and shape (e.g., Purple squares) indicate the same influence. The thicker the line and/or larger the dot, the more scale of export quantity.

Figure 3a shows that the United States account for 22.14% of world exports, has trade links with more than 170 countries, and has absolute influence in the CTN. India and Thailand have contacts with more than 160 countries; Argentina, Russia, and Ukraine each account for about 10% of world exports, with an out-degree between 100 and 140; the out-degree of France, Brazil, and Canada is similar to the previous echelon, while the export quantity is slightly lower (20–30 million tons). The export quantity of eight countries, including Italy, is less than 0.1 million tons, but the out-degree is higher than 90 (Table 3). The above-mentioned countries can have a huge share in the export market or have extensive trade links, have a pivotal position in the CTN, and can become the core node countries of the CNT.

Figure 3b shows that Mexico, Egypt, and Japan each account for about 5% of the world’s imports. Cereals imports are large and originate from relatively single sources, making them highly dependent on certain countries. South Korea and three other countries (Indonesia, Vietnam, and Algeria) imported more than 10 million tons, but the in-degree is low. Due to their large import quantity, it is more difficult for these countries to find alternative countries that can meet their import demands when there are problems with cereals exports from their main source countries. Therefore, such countries face a higher external supply risk. Under the pandemic influence, when it is difficult for these countries to obtain cereals from their major import sources and they have to reconsider their import partners in the global market, it is very likely that would trigger fluctuations in the CTN.

Important Betweenness centrality in the CTN plays a key role in the stability of the entire network. Figure 3c shows that the USA degree and BC value rank first in the world. France and the other three countries (Italy, India, and Thailand) have a secondary position in the influence on the CTN. Canada and the other 15 countries have a low degree but high BC value. In particular, the UAE and the Netherlands do not have high food production, but they are also important hubs in the CTN. The pandemic may lead to a decline in the efficiency or loss of functions of the food processing, transportation, and other connections in such hub countries, causing a decline in the efficiency of the entire trade network or even its collapse.

### 3.2. Community Structure and Analysis

**Figure 4:**
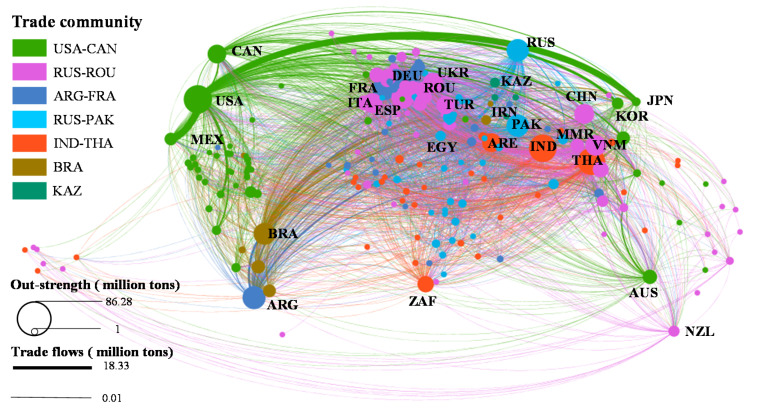
Community structure and flow patterns in the CTN. The same color indicates the same trade community. The thicker and larger the line and dot, the more scale of export quantity.

Analysis of the community structure shows that the CTN has formed seven communities that are dominated by major cereals exporting countries. The total quantity of trade within the community is close to 200 million tons, which is 51.31% of the global trade quantity. The trade quantity of the USA-CAN community accounts for 22.45% globally (Table 4). Cereals mainly flow to Japan, Mexico, South Korea, Colombia, Taiwan, and other Latin American and East Asian countries or regions (Figure 4 and Figure S1). More than 7% of imported cereals in the USA come from Canada. The trade quantity of the UKR-ROU community accounts for 12.46% globally, covering most countries in Europe and Africa. Cereals mainly flow to Europe and sub-Saharan Africa. The trade quantity of the ARG-FRA community accounts for 5.88% globally, and the cereals mainly flow to Northern and Western Europe and sub-Saharan Africa. The trade quantity of the RUS-PAK community accounts for 5.79% globally. The cereals mainly flow to countries in Africa and Southern Europe, such as Egypt, Bangladesh, Sudan, Nigeria, Yemen, Lebanon, etc. The IND-THA community has the most extensive trade links, with a trade quantity of 2.08% globally. Cereals mainly flow to the countries of sub-Saharan Africa and West Asia, such as South Africa, Benin, Cameroon, etc. The trade quantity of the BRA community accounts for 2.00% globally, and the trade links are mainly with the Latin American countries. Finally, the trade quantity of the KAZ community accounts for only 0.66% globally (Table 4), mainly in the internal flows among the countries in Central Asia (Figure 4).

From a perspective of dependence, the USA-CAN community is absolutely dependent (i.e., 80–100%) on the largest number of countries, of which 22 countries are absolutely dependent on the USA (Figure S2). In the remaining communities, with the exception of the BRA community and the ARG-FRA community that did not form an absolute dependency relationship, there are still 7–8 pairs of absolute dependencies in other communities. There is a direct relationship between trade dependence between countries and the concentration of cereals imports. Strong dependence will lead to a higher concentration of imports, and the external supply of cereals in importing countries is more susceptible to the risk of a single country COVID-19. On the other hand, if more countries in a community form an absolute dependence on a single country, then when the core exporting country has a higher risk of the pandemic, it is more likely to lead to an increased risk of external cereals supply for the members of the community (Figures S1 and S2).

### 3.3. Risk Simulation and Analysis

#### 3.3.1. Spatial Pattern of Risk Factors

**Figure 5:**
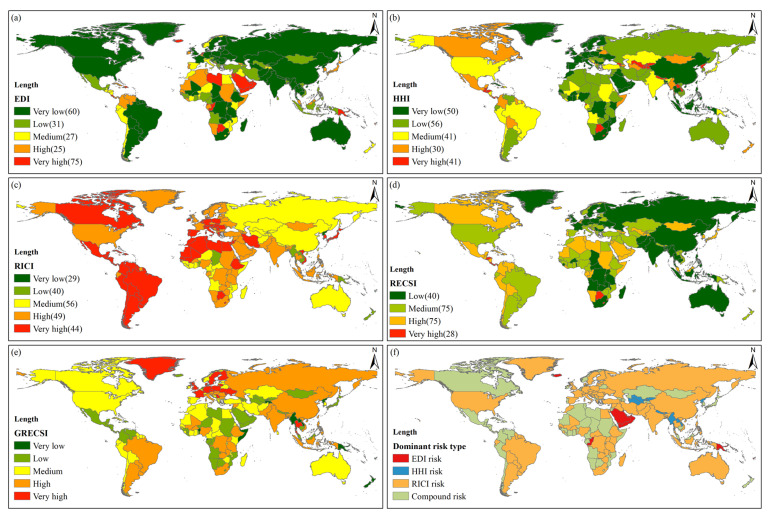
Spatial pattern of global cereals security risks and dominant risk types. (**a**) EDI; (**b**) HHI; (**c**) RICI; (**d**) RECSI; (**e**) GRECSI; (**f**) dominant risk type.

The result of the EDI analysis shows that nearly 60% of the countries (128 in total) in the world belong to the medium and above (high and very high) risk level (Figure 5a). Seventy-five countries are at a very high risk level, including 38 net food-importing developing countries (NFIDC). There are 25 and 27 countries with high and medium risk levels, respectively, which are mainly distributed in West Asia, North Africa, Latin America, and Southeast Asia, as well as Japan and South Korea. There are 91 countries with low and very low risk levels, including 39 NFIDC. The results of the HHI analysis show that approximately 50% of the countries are at the above-average risk level. Forty-one countries belong to a very high risk level, while 30 and 41 countries are at high and medium risk levels, respectively, scattered in Latin America, Central and Southeast Asia, and other regions (Figure 5b). The results of RICI analysis show that almost 70% of the countries are at a medium or higher risk level. Forty-four countries are at a very high risk level, of which 11 countries import cereals from a single country. There are also 49 and 56 countries with high and medium risk levels, respectively, and are mainly located in the Americas, Africa, Europe, and South and Southeast Asia (Figure 5c).

#### 3.3.2. Spatial Pattern of Comprehensive Risk

Affected by the pandemic, the RECSI of different countries around the world has increased to varying degrees. From a global perspective, the average RECSI value increased from 10.94 before COVID-19 to 18.08 during COVID-19, which is an increase of 65%. From a community perspective, the average RECSI of the USA-CAN community is the highest, and it is higher than the global average. The RECSI of the BRA community and the ARG-FRA community ranks second and third, respectively. The RECSI growth rates of the BRA, ARG-FRA, and RUS-PAK communities ranked among the top three, and all were higher than the global growth rates, increasing by 107%, 87%, and 78%, respectively (Table 4).

From the country perspective, the number of countries at five different risk levels (from high to low risk) is 28, 75, 75, 40, and 0, respectively. Among them, the countries with a very high risk level are mainly the Pacific island countries and some countries in Latin America and Africa. Countries with high and medium risk levels are mainly located in Latin America, North Africa, West, Central, and Southeast Asia. Among the top 20 countries in terms of import quantity, seven countries (e.g., Japan, Mexico, and South Korea) are at a high risk level, eight countries (e.g., Egypt, Spain, and Vietnam) are at a medium risk level, and the remaining countries are at a low risk level. Among the 45 NFIDC, 16 and 19 countries are at very high and high risk levels, respectively (Figure 5d).

By comparing the increase in RECSI after the pandemic outbreak and when there is no pandemic, it can be found that 16 countries are at a very high risk level, while 29 and 56 countries are at high and medium risk levels (Figure 5e). Regarding spatial distribution, Europe is characterized with the countries that have very high and high risk levels, East, Southeast, and South Asia are dominated by countries with high risk levels, South America is dominated by countries with medium and high risk levels, and Latin America, West, and Central Asia are dominated with countries that have low risk level. Among the top 20 countries in terms of import quantity, Germany has the highest GRESCI and belongs to the very high risk level. Seven countries, including China, Indonesia, and Brazil, also show significant growth and belong to the high risk level. The RECSI of NFIDC is relatively high, and under the influence of the pandemic, the RECSI increased insignificantly. Especially, and the GRESCI of most NFIDC is at low and very low levels.

### 3.4. Type of External Supply Risk Identity

The results of the dominant risk identification show that the number of countries with a compound risk type reached 108, accounting for nearly 50% of all countries, which are generally distributed in Latin America and Africa. The RECSI of these countries is generally high, and most of them are at medium or higher risk levels. Therefore, a single risk can cause serious external supply risks.

There are 73 countries that belong to the RICI risk type, which makes up 33% of all countries. Most of them are located in South America, Asia, and Europe, covering most of the world’s major cereals exporting countries. Eighteen of the top 20 exporting countries fall into this category. The RECSI of these countries was below 10 before the pandemic outbreak, and the original RECSI was relatively low. Following the COVID-19 outbreak, the risk of the pandemic in the country of origin of food imports became the main factor in the increase in the RECSI.

There are 29 countries that belong to the EDI risk type, accounting for 13% of all countries. They are mainly distributed in West Asia, including most of the island countries. The initial risk index of these countries is between 12 and 18, and the risk index after the COVID-19 outbreak is between 17 and 20. Basically, countries with the EDI risk type have limited food production capacities, food supply depends on imports, and the import sources are relatively scattered. External dependence is the dominant risk factor of the external cereals supply.

There are eight countries belonging to the HHI risk type, mainly located in Central, South, and Southeast Asia. The external supply of cereals in these countries is generally at medium risk. There is a relatively single import source of cereals, the main source of cereals is highly dependent on imports, and food imports are susceptible to factors such as the country’s food policy.

In addition, among the top 20 countries in terms of import quantity, countries with medium and low risk levels of RECSI are mostly dominated by the RICI risk, while countries with high risk levels of RECSI are mostly dominated by compound risks. In the NFIDC, 27 countries are dominated by the compound risks, while 18 countries are dominated by the EDI risk (Figure 5f).

## 4. Discussion

This study starts from the perspective of a complex network, uses FAOSTAT global data on main cereals from 2016 to 2018, and identifies the core nodes for the stable operation of the CTN based on the complex network method, and then analyzes the main cereals flow pattern and interdependence. Next, based on the trade perspective, a global external supply risk assessment model was built that includes the risk factors such as the RICI, and then the global, community, and country-level changes in external supply risk, and the types of dominated risks affected by the pandemic were analyzed.

The main contributions are: (1) a simple and synthetical method, namely the risk of global external cereal supply index (RECSI) by integrating EDI, HHI, RICI, was developed to detect global cereals security risks; (2) analysis of the changes and spatial pattern of the global RECSI under the influence of pandemic; and (3) identification of the dominant types of global external supply risk under the influence of pandemic. The study provides a scientific basis for the countries with different dominant risk types to implement policies that address the external risks of cereals supply. The novelty is that, unlike previous studies that took into account risk factors such as political stability and spatial distance to assess the risk of external supply of resources [29], this research is based on the increased probability of export controls and trade interruptions in the context of a global pandemic. The inclusion of the epidemiological risk of the import source country into the external food supply risk assessment is closer to reality. However, the reliability of the COVID-19 Risk Index (http://covid19-risk-index.com/, accessed on 1 February 2021) could affect the evaluation of the RICI. In particular, the mean value of the COVID-19 Risk Index may cover up or obscure the temporal processes and results of the RECSI. We found that the external supply risk of main cereals exporting countries is dominated by the RICI risk. Therefore, maintaining the stability of the original trade policy rather than adopting trade restrictions is more likely to maintain their external supply risk. This confirms the need to avoid the implementation of the trade control measures.

The distribution of global CTN degree values shows a typical scale-free feature. The great heterogeneity of the trade network means that the removal of the highly connected nodes will lead to a rapid increase in the network diameter and the collapse of the network [38]. Existing studies have shown that scale-free networks are robust to random failures while they show high vulnerability to malicious attacks [32]. As a result, the core nodes in the CTN have become the key points of potential risks in CTN. In fact, cereals production is a basic factor in cereals flow, and the patterns of cereals production and demand basically lead to the creation of the dominant features of the global CTN. From 2016 to 2018, the global (184 countries) production of main cereals reached 2093.09 million tons per year, and the top 20 countries accounted for about 80% of the production. On the other hand, the global per capita main cereals occupancy is about 280 kg/person, with more than 70% of countries being below the global average and 60% of the countries’ per capita occupancy is less than 150 kg/person on an annual level. The dislocation between the geographical area of food consumption and the geographical area of food production is an important reason for trade demand. There are more than 20% of countries that rely entirely on the international market for their supply of the main cereals, and almost one-third of the countries are more than 90% dependent on external sources. Due to the impact of the pandemic, among the 20 core countries that account for 75% and 81% of the global food production and export, respectively, eight countries have issued trade restriction policies [39], and 15 countries have CRI above 50 (Table 5 and Figure S3). Uncertainty in the trade policies of these core exporting countries and the uncertainty in the pandemic crisis has become a source of risks to the global external supply of cereals, and these countries need to focus on maintaining the stability of the global CTN.

Affected by the pandemic, the global RECSI increased by 65%. The USA-CAN community has the highest risk index, while the BRA community and the AGR-FRA community have the largest increase in the RECSI of the seven communities. In addition, 60%, 50%, and 70% of countries face medium and above (high and very high) EDI risk, HHI risk, and RICI risk, respectively. Countries with high RECSI values are mainly Pacific island countries, Latin American and African countries, but also include Japan, Mexico, and South Korea with large imports. About 80% of NFIDCs are at high or very high RECSI levels, while the increase in RECSI in European countries is generally at a high level. Furthermore, about 50% of the countries are characterized by compound risks. NFIDC is dominated by compound risks and EDI risks. Many net cereals exporting countries are characterized by the RICI risk, and the instability of the cereals imports caused by the pandemic will exacerbate the instability of cereals export policies, causing fluctuations in the global cereals market.

The global CTN stability is potentially under the threat of trade disruption. Since the World Health Organization announced the COVID-19 pandemic, more than 20 countries have introduced restrictions on food exports [39], and the global CTN has also been locally disrupted. Restrictions on the movement of people, poor operation of the cereals production and processing chain, and the delayed arrival of goods in ports threaten the normal functioning of the global CTN. These problems have been addressed in previous studies on epidemics and food security [40], and early studies on the impacts of the pandemic have also confirmed the above judgments. During the most severe period of the pandemic in China, from January to February 2020, the export quantity of labor-intensive products, rice, and peanuts, fell by 18% and 31%, respectively [11]. In addition, to prevent the spread of COVID-19, more than 120 countries and regions have prohibited or restricted the entry of ships, and there are also a large number of crew members who refuse to go to countries with severe pandemic situations due to fear of virus infection. Accordingly, the world is experiencing the most serious transportation crisis in decades.

**Figure 6:**
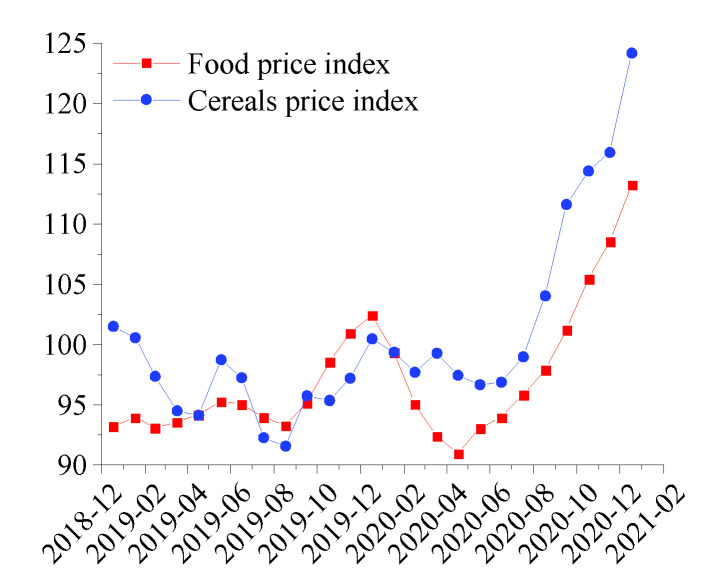
The global food price index from January 2019 to January 2021.

The continuous rise in food prices is becoming a key factor influencing the external supply risks. As of January 2021, the cereal price index has risen for seven consecutive months, reaching 124.2 points, which is an increase of 22% compared to its highest point in 2019, reflecting an increasingly tight global supply [41]. The 2008 food price crisis showed that concerns about food supply policies could easily escalate into a price [42]. The export ban at the beginning of the pandemic shows many similarities with the above-mentioned food price crisis, which is worth worrying about (Figure 6).

The results of our study are in line with the results previously released by the World Bank. Countries with higher external supply risks are generally developing countries and the least developed economies, and the countries most affected are those with high dependence on imported food [11]. Food and Agriculture Organization of the United Nations (FAO) estimates that 45 countries need external assistance and that the decline in income caused by pandemic has become an important factor affecting global food security [43]. Overall, the pandemic continues to develop, the economic recovery is still unclear, and the consequences of domestic food production due to the pandemic have not yet been fully manifested. In addition, the efficiency of the port transportation system and the decline in economic affordability have led to the “interruption” of the global external supply of food. There are several potential threats, such as the “price crisis” and the “payment dilemma”, and multiple food security risks remain widespread [42].

Based on this study, we can develop and propose policy recommendations to maintain the stability of the global food trade network and reduce the risk of external food supply from the two dimensions of liquidity and concentration. Given the fact that the external supply risk of the important cereals exporting countries is dominated by the risk of the pandemic situation in their importing partner countries, it is necessary to maintain the liquidity of the global CTN, call for avoiding the restrictive trade policies and prevent the trade restrictions of core countries in the CTN. This is extremely important for maintaining global food security. Countries that are dominated by the EDI risk need to stabilize and improve their domestic food supply levels. Countries that are dominated by the HHI and the RICI risk should consider opening new sources of imports across communities and regions to achieve diversification of cereals import channels. In the long run, improving the domestic production capacity of cereals is a fundamental measure, and increased food aid is now even more necessary and urgent [44]. In addition, several factors could affect cereals production, which involves climate [45] and new smart agriculture technology [46].

As for future research, the impact of the pandemic on global food security is multi-faceted and multi-dimensional, such as cereals supply chain [4] and food resilience [47]. In fact, with the growth of population, acceleration of urbanization, and occurrence frequency of pandemic disease, the question of the cereals supply chain will become more and more complex and poignant. This study only assessed the food security risks of different countries from the trade perspective and the perspective of external supply. Future research can be expanded to comprehensively assess the impact of the pandemic on the stability of internal and external food supply, to assess the impact of the pandemic on global food security, and to propose a systematic response strategy.

## Figures and Tables

**Table 1:** Criteria for classification of different risk levels.

Level	Natural Breaks				Equal Interval
	R_EDI_	R_HHI_	R_RICI_	GRECSI	RECSI
Very Low	1.00–1.14	1.08–1.21	1.19–1.38	0.05–0.40	1–6
Low	1.14–1.38	1.21–1.37	1.38–1.51	0.40–0.77	7–12
Medium	1.38–1.62	1.37–1.56	1.51–1.59	0.77–1.33	13–18
High	1.62–1.85	1.56–1.80	1.59–1.66	1.33–2.25	19–24
Very high	1.85–2.00	1.80–2.00	1.66–1.74	2.25–5.00	25–30

**Table 2:** Criteria for classification into dominant risk factors.

Classification Criteria	Type Name
CR_DEI_ > 50%	EDI risk
CR_HHI_ > 50%	HHI risk
CR_RICI_ > 50%	RICI risk
CR_DEI_, CR_HHI_, CR_RICI_ are all < 50%	Compound risks

**Table 3:** Characteristics of the main cereals exporting and importing nations worldwide.

Rank	ISO3	Export		Export Degree (# Nations)	ISO3	Import		Import Degree (# Nations)	Production		
		Quantity (Million Tons)	Proportion (%)			Quantity (Million Tons)	Proportion (%)		ISO3	Quantity (Million Tons)	Proportion (%)
1	USA	86.28	22.14	173	JPN	22.20	5.70	39	CHN	439.90	21.02
2	RUS	39.29	10.08	127	EGY	21.09	5.41	31	USA	321.24	15.35
3	UKR	36.57	9.39	110	MEX	20.64	5.30	23	IND	236.55	11.30
4	ARG	35.86	9.20	133	KOR	14.39	3.69	39	RUS	87.35	4.17
5	BRA	25.68	6.59	124	ESP	13.85	3.55	62	BRA	71.54	3.42
6	CAN	23.36	6.00	100	VNM	13.27	3.41	40	ARG	61.23	2.93
7	FRA	22.44	5.76	132	ITA	12.74	3.27	71	IDN	55.83	2.67
8	AUS	16.28	4.18	66	IDN	12.54	3.22	26	UKR	55.21	2.64
9	IND	10.84	2.78	167	DZA	12.44	3.19	41	FRA	47.67	2.28
10	ROU	10.16	2.61	86	NLD	11.09	2.85	72	CAN	45.25	2.16
11	THA	9.98	2.56	165	IRN	9.85	2.53	36	BGD	39.97	1.91
12	DEU	8.29	2.13	108	CHN	9.85	2.53	46	PAK	39.08	1.87
13	BGR	5.53	1.42	56	BRA	8.87	2.28	26	VNM	32.21	1.54
14	HUN	5.45	1.40	58	SAU	8.46	2.17	49	MEX	31.34	1.50
15	KAZ	5.04	1.29	33	BGD	8.00	2.05	25	TUR	27.31	1.30
16	POL	4.09	1.05	71	PHL	7.89	2.02	35	DEU	27.04	1.29
17	PAK	3.71	0.95	127	TUR	7.88	2.02	63	ROU	24.14	1.15
18	VNM	3.63	0.93	115	DEU	7.54	1.93	69	THA	23.31	1.11
19	PRY	2.80	0.72	59	COL	7.06	1.81	24	PHL	20.08	0.96
20	CZE	2.72	0.70	34	MAR	6.85	1.76	37	MMR	19.08	0.91
/	Total	357.98	91.88	/	Total	236.49	60.70	/	Total	1705.36	81.48
/	Global	389.71	100	/	Global	389.71	100	/	Global	2093.09	100

Note: ISO3 is the nation code. See Table S1 for the full country name.

**Table 4:** Characteristics of the CTN community structure.

Community	Intra-Community Trade		Node		Edge		RECSI		
	Quantity (Million tons)	Proportion (%)	Number	Proportion (%)	Number	Proportion (%)	Before COVID-19	The Pandemic	GRECSI
USA-CAN	87.48	22.45	50	22.94	294	5.74	13.92	22.50	62
UKR-ROU	48.54	12.46	61	27.98	777	15.18	10.33	16.74	62
ARG-FRA	22.90	5.88	24	11.01	122	2.38	9.21	17.21	87
RUS-PAK	22.58	5.79	37	16.97	174	3.40	9.14	16.30	78
IND-THA	8.1	2.08	35	16.06	126	2.46	11.46	16.91	48
BRA	7.79	2.00	7	3.21	14	0.27	8.71	18.00	107
KAZ	2.57	0.66	4	1.83	10	0.20	9.50	15.50	63
Global	389.71	100	218	100	5118	100	10.94	18.08	65

**Table 5:** CRI and trade restriction policy at the core node of the CTN.

Country	BC Node and/or Out-Degree Node	CRI	Trade Restriction	Country	BC Node and/or Out-Degree Node	CRI	Trade Restriction
USA	Both	68.61	No	PAK	Both	48.85	Yes
RUS	Both	56.14	Yes	VNM	Out−Degree Node	31.83	Yes
UKR	Both	68.20	Yes	ZAF	BC Node	65.15	No
ARG	Both	73.95	YES	GBR	BC Node	64.10	No
BRA	Both	71.52	No	CHN	Both	26.53	No
CAN	Both	54.12	No	NLD	BC Node	67.89	No
FRA	Both	68.68	No	ITA	Both	63.21	No
IND	Both	48.30	Yes	ESP	Both	62.59	No
THA	Both	33.25	Yes	ARE	Both	45.37	No
DEU	Both	54.35	No	TUR	Both	51.42	Yes

## Data Availability

The data presented in this study are available on request to authors.

## Supplementary Materials

The following are available online at https://www.mdpi.com/2304-8158/10/6/1168/s1, Figure S1: Flow of the main cereals within the global CTN communities, Figure S2: Statistics on the level of dependence on a single country in different communities, Figure S3: Spatial pattern of (a) trade community of cereals trade networks (CTN) and (b) the COVID-19 Risk index (CRI), Table S1: Country (218 in total) name and ISO3 code.
