# Supplementary material for: Risk of Global External Cereals Supply under the Background of the COVID-19 Pandemic: Based on the Perspective of Trade Network
Source: Foods. 2021 May 23;10(6):1168. doi: 10.3390/foods10061168 (PMC8246323; doi:10.3390/foods10061168)
Supplement: Supplementary file 1 [file foods-10-01168-s001.zip › foods-1211022-supplementary.pdf]

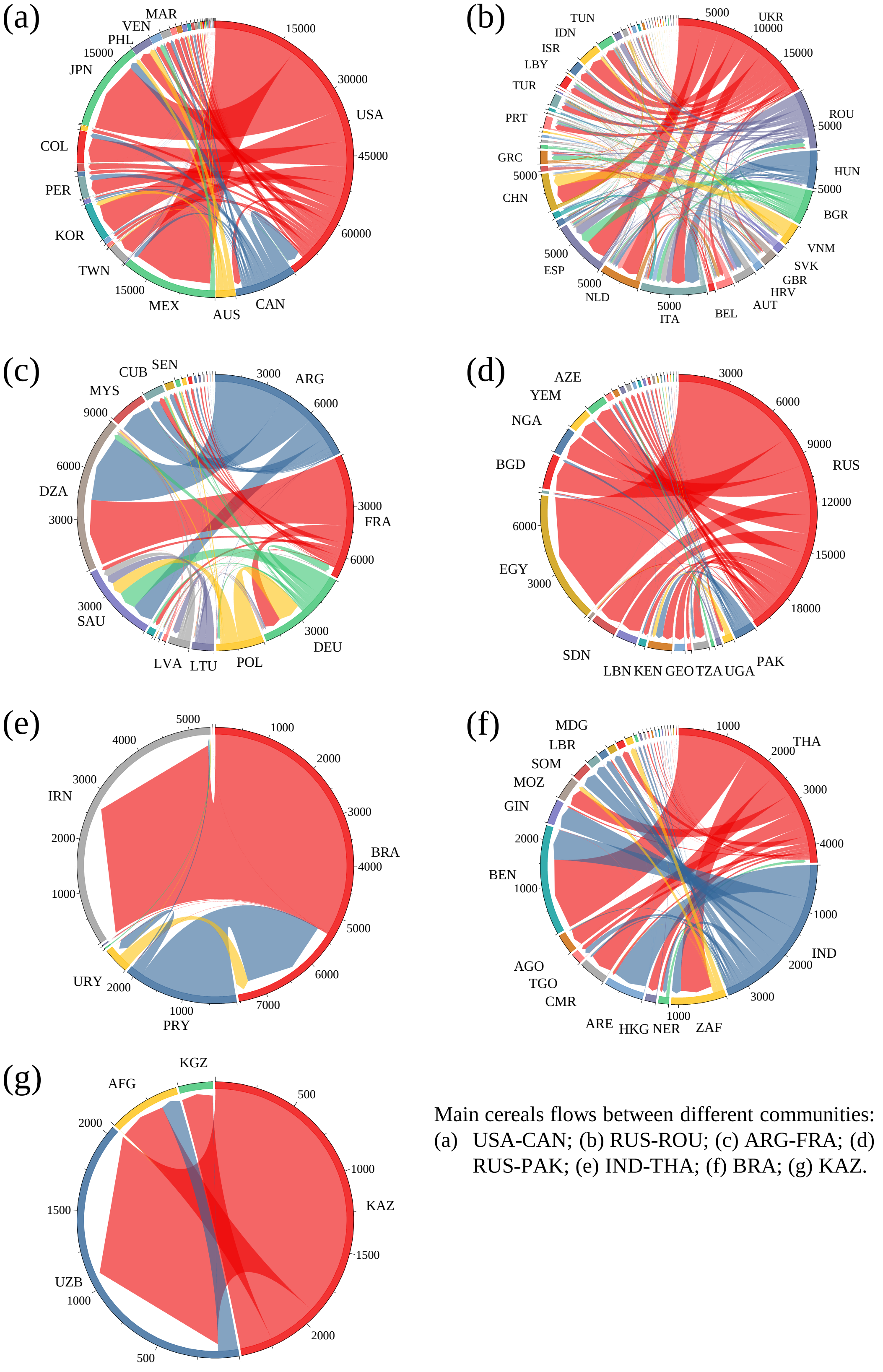

Figure S1 Flow of the main cereals within the global CTN communities. A total of 218 countries are ranked according to the total trade quantity and plotted clockwise in descending order. The size of the out bar indicates the total trade quantity (unit: 1000t). Export quantity is indicated with links emanating from the outer bar of the same color.

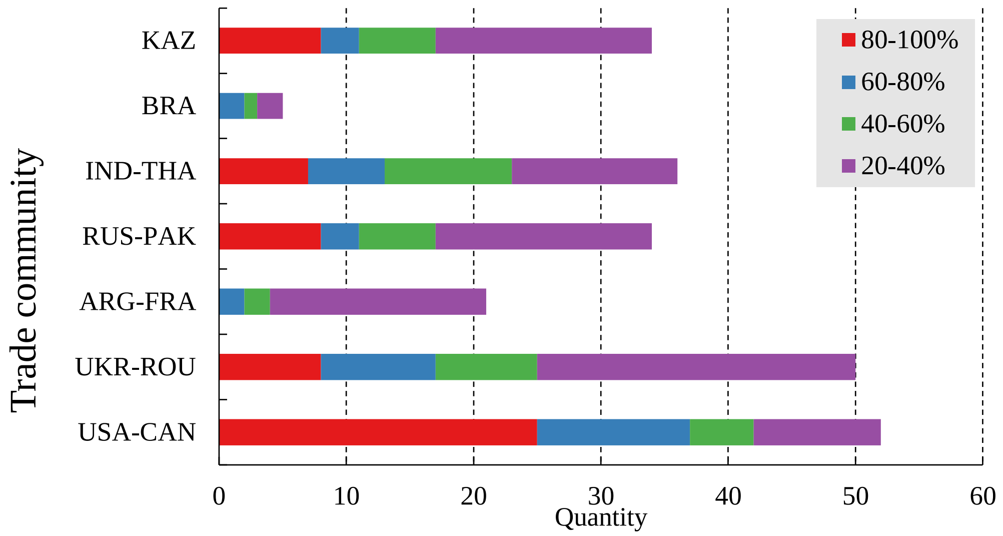

Figure S2 Statistics on the level of dependence on a single country in different communities.

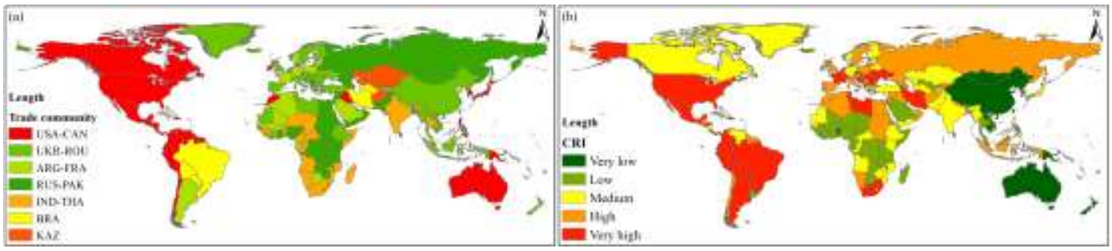

Figure S3 Spatial pattern of (a) trade community of cereals trade networks (CTN) and (b) the COVID-19 Risk Index **(CRI)**

Table S1 Country (218 in total) name and ISO3 code

SN	ISO3	Country	SN	ISO3	Country	SN	ISO3	Country
1	ABW	Aruba	25	BLR	Belarus	49	CXR	Christmas Island
2	AFG	Afghanistan	26	BLZ	Belize	50	CYM	Cayman Islands
3	AGO	Angola	27	BMU	Bermuda	51	CYP	Cyprus
4	AIA	Anguilla	28	BOL	Bolivia	52	CZE	Czech Republic
5	ALB	Albania	29	BRA	Brazil	53	DEU	Germany
6	AND	Andorra	30	BRB	Barbados	54	DJI	Djibouti
7	ANT	Netherlands Antilles	31	BRN	Brunei Darussalam	55	DMA	Dominica
8	ARE	United Arab Emirates	32	BTN	Bhutan	56	DNK	Denmark
9	ARG	Argentina	33	BWA	Botswana	57	DOM	Dominican Republic
10	ARM	Armenia	34	CAF	Central African Republic	58	DZA	Algeria
11	ASM	American Samoa	35	CAN	Canada	59	ECU	Ecuador
12	ATG	Antigua and Barbuda	36	CHE	Switzerland	60	EGY	Egypt
13	AUS	Australia	37	CHL	Chile	61	ERI	Eritrea
14	AUT	Austria	38	CHN	China	62	ESP	Spain
15	AZE	Azerbaijan	39	CIV	Cote d'Ivoire	63	EST	Estonia
16	BDI	Burundi	40	CMR	Cameroon	64	ETH	Ethiopia
17	BEL	Belgium	41	COD	Democratic Republic of the Congo	65	FIN	Finland
18	BEN	Benin	42	COG	Congo	66	FJI	Fiji
19	BFA	Burkina Faso	43	COK	Cook Islands	67	FLK	Falkland Islands (Malvinas)
20	BGD	Bangladesh	44	COL	Colombia	68	FRA	France
21	BGR	Bulgaria	45	COM	Comoros	69	FRO	Faroe Islands
22	BHR	Bahrain	46	CPV	Cape Verde	70	FSM	Micronesia, Federated States of
23	BHS	Bahamas	47	CRI	Costa Rica	71	GAB	Gabon
24	BIH	Bosnia and Herzegovina	48	CUB	Cuba	72	GBR	United Kingdom

SN	ISO3	Country	SN	ISO3	Country	SN	ISO3	Country
73	GEO	Georgia	98	JAM	Jamaica	123	MDV	Maldives
74	GHA	Ghana	99	JOR	Jordan	124	MEX	Mexico
75	GIN	Guinea	100	JPN	Japan	125	MHL	Marshall Islands
76	GMB	Gambia	101	KAZ	Kazakhstan	126	MKD	The Republic of North Macedonia
77	GNB	Guinea-Bissau	102	KEN	Kenya	127	MLI	Mali
78	GNQ	Equatorial Guinea	103	KGZ	Kyrgyzstan	128	MLT	Malta
79	GRC	Greece	104	KHM	Cambodia	129	MMR	Burma
80	GRD	Grenada	105	KIR	Kiribati	130	MNE	Montenegro
81	GRL	Greenland	106	KNA	Saint Kitts and Nevis	131	MNG	Mongolia
82	GTM	Guatemala	107	KOR	Korea, Republic of	132	MNP	Northern Mariana Islands
83	GUM	Guam	108	KWT	Kuwait	133	MOZ	Mozambique
84	GUY	Guyana	109	LAO	Lao People's Democratic Republic	134	MRT	Mauritania
85	HKG	Hong Kong	110	LBN	Lebanon	135	MSR	Montserrat
86	HND	Honduras	111	LBR	Liberia	136	MUS	Mauritius
87	HRV	Croatia	112	LBY	Libyan Arab Jamahiriya	137	MWI	Malawi
88	HTI	Haiti	113	LCA	Saint Lucia	138	MYS	Malaysia
89	HUN	Hungary	114	LKA	Sri Lanka	139	NAM	Namibia
90	IDN	Indonesia	115	LSO	Lesotho	140	NCL	New Caledonia
91	IND	India	116	LTU	Lithuania	141	NER	Niger
92	IRL	Ireland	117	LUX	Luxembourg	142	NFK	Norfolk Island
93	IRN	Iran (Islamic Republic of)	118	LVA	Latvia	143	NGA	Nigeria
94	IRQ	Iraq	119	MAC	Macau	144	NIC	Nicaragua
95	ISL	Iceland	120	MAR	Morocco	145	NIU	Niue
96	ISR	Israel	121	MDA	Republic of Moldova	146	NLD	Netherlands
97	ITA	Italy	122	MDG	Madagascar	147	NOR	Norway

SN	ISO3	Country	SN	ISO3	Country	SN	ISO3	Country
148	NPL	Nepal	173	SLB	Solomon Islands	171	SGP	Singapore
149	NRU	Nauru	174	SLE	Sierra Leone	172	SHN	Saint Helena
150	NZL	New Zealand	175	SLV	El Salvador	198	TUR	Turkey
151	OMN	Oman	176	SOM	Somalia	199	TUV	Tuvalu
152	PAK	Pakistan	177	SPM	Saint Pierre and Miquelon	200	TWN	Taiwan
153	PAN	Panama	178	SRB	Serbia	201	TZA	United Republic of Tanzania
154	PER	Peru	179	SSD	South Sudan	202	UGA	Uganda
155	PHL	Philippines	180	STP	Sao Tome and Principe	203	UKR	Ukraine
156	PLW	Palau	181	SUR	Suriname	204	URY	Uruguay
157	PNG	Papua New Guinea	182	SVK	Slovakia	205	USA	The United States
158	POL	Poland	183	SVN	Slovenia	206	UZB	Uzbekistan
159	PRK	Korea, Democratic People's Republic of	184	SWE	Sweden	207	VAT	Holy See (Vatican City)
160	PRT	Portugal	185	SWZ	Swaziland	208	VCT	Saint Vincent and the Grenadines
161	PRY	Paraguay	186	SYC	Seychelles	209	VEN	Venezuela
162	PSE	Palestine	187	SYR	Syrian Arab Republic	210	VGB	British Virgin Islands
163	PYF	French Polynesia	188	TCA	Turks and Caicos Islands	211	VNM	Viet Nam
164	QAT	Qatar	189	TCD	Chad	212	VUT	Vanuatu
165	ROU	Romania	190	TGO	Togo	213	WLF	Wallis and Futuna Islands
166	RUS	Russia	191	THA	Thailand	214	WSM	Samoa
167	RWA	Rwanda	192	TJK	Tajikistan	215	YEM	Yemen
168	SAU	Saudi Arabia	193	TKM	Turkmenistan	216	ZAF	South Africa
169	SDN	Sudan	194	TLS	Timor-Leste	217	ZMB	Zambia
170	SEN	Senegal	195	TON	Tonga	218	ZWE	Zimbabwe

Note: SN is abbreviation of serial number.
